# Symptom Prediction and Mortality Risk Calculation for COVID-19 Using Machine Learning

**DOI:** 10.3389/frai.2021.673527

**Published:** 2021-06-22

**Authors:** Elham Jamshidi, Amirhossein Asgary, Nader Tavakoli, Alireza Zali, Farzaneh Dastan, Amir Daaee, Mohammadtaghi Badakhshan, Hadi Esmaily, Seyed Hamid Jamaldini, Saeid Safari, Ehsan Bastanhagh, Ali Maher, Amirhesam Babajani, Maryam Mehrazi, Mohammad Ali Sendani Kashi, Masoud Jamshidi, Mohammad Hassan Sendani, Sahand Jamal Rahi, Nahal Mansouri

**Affiliations:** ^1^ Functional Neurosurgery Research Center, Shohada Tajrish Comprehensive Neurosurgical Center of Excellence, Shahid Beheshti University of Medical Sciences, Tehran, Iran; ^2^ Department of Biotechnology, College of Sciences, University of Tehran, Tehran, Iran; ^3^ Trauma and Injury Research Center, Iran University of Medical Sciences, Tehran, Iran; ^4^ Department of Clinical Pharmacy, School of Pharmacy, Shahid Beheshti University of Medical Sciences, Tehran, Iran; ^5^ School of Mechanical Engineering, Sharif University of Technology, Tehran, Iran; ^6^ School of Electrical and Computer Engineering, Engineering Faculty, University of Tehran, Tehran, Iran; ^7^ Department of Genetic, Faculty of Advanced Science and Technology, Tehran Medical Sciences, Islamic Azad University, Tehran, Iran; ^8^ Department of Anesthesiology, Tehran University of Medical Sciences, Tehran, Iran; ^9^ School of Management and Medical Education, Shahid Beheshti University of Medical Sciences, Tehran, Iran; ^10^ Department of Pharmacology, School of Medicine, Shahid Beheshti University of Medical Sciences, Tehran, Iran; ^11^ Department of Business Management, Faculty of Management, University of Tehran, Tehran, Iran; ^12^ Department of Exercise Physiology, Tehran University, Tehran, Iran; ^13^ Department of Computer Engineering, Sharif University of Technology, Tehran, Iran; ^14^ Laboratory of the Physics of Biological Systems, Institute of Physics, École Polytechnique Fédérale de Lausanne (EPFL), Lausanne, Switzerland; ^15^ Division of Pulmonary Medicine, Department of Medicine, Lausanne University Hospital (CHUV), University of Lausanne (UNIL), Lausanne, Switzerland; ^16^ Swiss Institute for Experimental Cancer Research (ISREC), School of Life Sciences, École Polytechnique Fédérale de Lausanne (EPFL), Lausanne, Switzerland; ^17^ Research Group on Artificial Intelligence in Pulmonary Medicine, Division of Pulmonary Medicine, Lausanne University Hospital (CHUV), Lausanne, Switzerland

**Keywords:** COVID-19, artificial intelligence, machine learning, symptom, mortality

## Abstract

**Background:** Early prediction of symptoms and mortality risks for COVID-19 patients would improve healthcare outcomes, allow for the appropriate distribution of healthcare resources, reduce healthcare costs, aid in vaccine prioritization and self-isolation strategies, and thus reduce the prevalence of the disease. Such publicly accessible prediction models are lacking, however.

**Methods:** Based on a comprehensive evaluation of existing machine learning (ML) methods, we created two models based solely on the age, gender, and medical histories of 23,749 hospital-confirmed COVID-19 patients from February to September 2020: a symptom prediction model (SPM) and a mortality prediction model (MPM). The SPM predicts 12 symptom groups for each patient: respiratory distress, consciousness disorders, chest pain, paresis or paralysis, cough, fever or chill, gastrointestinal symptoms, sore throat, headache, vertigo, loss of smell or taste, and muscular pain or fatigue. The MPM predicts the death of COVID-19-positive individuals.

**Results:** The SPM yielded ROC-AUCs of 0.53–0.78 for symptoms. The most accurate prediction was for consciousness disorders at a sensitivity of 74% and a specificity of 70%. 2,440 deaths were observed in the study population. MPM had a ROC-AUC of 0.79 and could predict mortality with a sensitivity of 75% and a specificity of 70%. About 90% of deaths occurred in the top 21 percentile of risk groups. To allow patients and clinicians to use these models easily, we created a freely accessible online interface at www.aicovid.net.

**Conclusion:** The ML models predict COVID-19-related symptoms and mortality using information that is readily available to patients as well as clinicians. Thus, both can rapidly estimate the severity of the disease, allowing shared and better healthcare decisions with regard to hospitalization, self-isolation strategy, and COVID-19 vaccine prioritization in the coming months.

## Introduction

The COVID-19 pandemic of the 2019 novel coronavirus (SARS-CoV-2) started in December 2019 and is spreading rapidly, with approximately 62.5 million confirmed cases and 1.5 million deaths by the end of November 2020 (WHO, 2020).

The severity of the disease varies widely between different patients, ranging from no symptoms to a mild flu-like illness, to severe respiratory symptoms, and to multi-organ failure leading to death. Among the symptoms, fever, cough, and respiratory distress are more prevalent than symptoms such as consciousness disorders and loss of smell and taste (Tabata et al., 2020; Jamshidi et al., 2021b). In general, complications are common among elderly patients and those with pre-existing conditions. The intensive care unit (ICU) admission rate is substantially higher for these groups (Abate et al., 2020; Jamshidi et al., 2021a).

The Center for Disease Control (CDC) and the World Health Organization (WHO) consider the identification of individuals at higher risk a top priority. This identification could be used for numerous solutions to moderate the consequences of the pandemic for the most vulnerable (CDC COVID-19 Response Team, 2020) as well as minimize the presence of actively ill patients in society.

This requires the prediction of the symptoms and mortality risk for infected individuals. While symptom prediction models exist for cancer, no such models have been designed for COVID-19 (Levitsky et al., 2019; Goecks et al., 2020). To make rapid, evidence-based decisions possible, they will ideally be based on readily available patient information, i.e., demographic attributes and past medical history (PMH) as opposed to costly laboratory tests. Early decision-making is critical for timely triage and clinical management of patients. For instance, clinical and laboratory data can only be assessed after presenting the individual to a health care center, increasing the risk of unnecessary exposures to the virus and increasing costs (Sun et al., 2020). These parameters are not available immediately and are partly subject to human error. Also, factors like genetic predisposition may increase the models’ accuracy but are not broadly available.

With the growth of big data in healthcare and the introduction of electronic health records, artificial intelligence (AI) algorithms can be integrated into hospital IT systems and have shown promise as computer-aided diagnosis and prognostic tools. In the era of COVID-19, AI has played an essential role in the early diagnosis of infection, the prognosis of hospitalized patients, contact tracing for spread control, and drug discovery (Lalmuanawma et al., 2020). AI methods can have a higher accuracy over classical statistical analyses.

In contrast to the few previously available COVID-19 risk scales, our mortality prediction model uses a selection of variables that are in principle accessible to all patients and thus can be used immediately after diagnosis (Assaf et al., 2020; Pan et al., 2020). This model not only has a significant benefit in early decision making in the hospital setting, but because it does not require clinicians or laboratories, it can serve as a triage tool for patients in an outpatient setting, in telemedicine, or as a self-assessment tool. For example, decisions on outpatient vs. inpatient care can be made remotely by estimating the most probable symptoms and severity risks. This lessens the strain on health care resources, unnecessary costs, and unwanted exposures to infected patients.

Here, we implemented 2 ML methods to predict the symptoms and the mortality of patients with COVID-19. Overall, 23,749 patients were included in the study. The predictors used for the models were age, sex, and PMH of the patients. Both of these models achieved predictions with high accuracy. To our knowledge, this is one of the largest datasets of COVID-19 cases and is the only study that uses patient-available data for the prediction of COVID-19 symptoms and mortality. Furthermore, this study is the most extensive study for mortality prediction for COVID-19 using ML-based on any set of predictors (An et al., 2020; Gao et al., 2020; Vaid et al., 2020; Yadaw et al., 2020).

We also created an online calculator where each individual can predict their COVID-19 related symptoms and risk (www.aicovid.net).

For a standardized representation of the methodology and results of this analysis an adapted version of the Transparent Reporting of Multivariable Prediction Model for Individual Prognosis or Diagnosis (TRIPOD) guideline was followed (Collins et al., 2015).

## Methods

### Source of Data and Participants

In this cohort study, we used the Hospital Information System (HIS) of 74 secondary and tertiary care hospitals across Tehran, Iran. The eligibility criteria were defined as confirmed or suspected SARS-CoV-2 infections of people aged 18–100 years registered in the referred HIS. The final database used to design the models was obtained by aggregating the 74 hospitals’ HIS. The study included patients referred to any of the hospitals between February 1, 2020, and September 30, 2020. Patients were followed up through October 2020 until all the registered patients had the specific death or survival outcome needed for the mortality prediction model (MPM). This study was approved by the Iran University of Medical Sciences Ethics Committee.

### Outcome

#### Symptom Prediction Model

The patients’ symptoms at the time of admission, as recorded in the HIS, were considered as the outputs of the Symptom Prediction Model (SPM). All stated symptoms were clustered in 12 categories to be predicted by the model. The groups are cough, loss of smell or taste, respiratory distress, vertigo, muscular pain or fatigue, sore throat, fever or chill, paresis or paralysis, gastrointestinal problems, headache, chest pain, and consciousness disorders.

### Mortality Prediction Model

Death or survival as per the HIS records was defined as the output of the mortality prediction model (MPM).

### Predictors

The patients’ age, sex, and past medical history (PMH), as detailed in Table 1, were used as predictors for both models. The selection of variables as predictors was based on the available recorded data. All these predictors were recorded in the HIS at the time of admission.

**TABLE 1:** List of predictors**.** Predictor variables for mortality risk and symptom prediction of COVID-19.

Category	Variable	Description
Demographic	Age	In years
	Sex	Male or female
Past/Current Medical Conditions	*Cancer*	Current chemotherapy, radiotherapy, immunotherapy, bone marrow or stem cell transplantation
	Liver disorders	Chronic hepatitis (type B or C), alcohol-related liver disease, primary biliary cirrhosis, primary sclerosing cholangitis, hemochromatosis, cirrhosis
	Blood disorders	Anemia (iron deficiency, thalassemia minor and major, sickle cell disease), coagulopathies (hemophilia and platelet disorders)
	Immune disorders	Immune deficiency (acquired immunodeficiency syndrome, treatment with steroids and immune suppressors), autoimmune disease (rheumatoid arthritis, systemic lupus erythematosus, ankylosing spondylitis, vasculitis).
	Cardiovascular disease	Congestive heart failure, cardiovascular events (myocardial infarction, stroke, angina), valvular heart disease, arrhythmia (e.g. atrial fibrillation)
	Kidney disorders	Chronic kidney disease (stage 3, 4, and end-stage renal disease)
	Respiratory disorders	Asthma, chronic obstructive pulmonary disease (emphysema and chronic bronchitis), extrinsic allergic alveolitis, cystic fibrosis, interstitial lung disease, sarcoidosis, bronchiectasis, pulmonary hypertension
	Neurological disorders	Epilepsy, Parkinson’s disease, motor neuron disease, cerebral palsy, dementia, multiple sclerosis
	Endocrine disorders	Hyperthyroidism, hypothyroidism, cushing syndrome, pheochromocytoma, thyroiditis, hyperaldosteronism
	Diabetes mellitus	Type 1 and type 2 diabetes, maturity onset diabetes of the young, insipidus, gestational diabetes
	Hypertension	Primary and secondary
	Psychiatric disorders (removed due to low prevalence)	Bipolar disorder, psychosis, schizophrenia, major depression disorder
	Thrombosis (removed due to low prevalence)	Venous thromboembolism, pulmonary thromboembolism

### Missing Data

We only included patients with the required data. Due to the absence of missing data, there was no imputation of missing values.

### Pre-Processing

Symptoms and predictor variables from the medical histories with an incidence of less than 0.2% were removed to reduce noise. This removed past COVID-19 infections, thrombosis, psychiatric disorders, and organ or bone marrow transplantation from the set of predictor variables. The removed symptoms were tachycardia, seizure, nasal congestion, and skin problems.

Sex, PMH, and symptoms were encoded as binary variables. In training and test sets, the only continuous predictor, age, was standardized to zero mean and unit standard deviation.

### Machine Learning Methods

To ensure generalizability, a 5-fold cross-validation algorithm was employed [Performance evaluation of classification algorithms by k-fold and leave-one-out cross validation, (Wong, 2015). All records were randomly separated into five independent subsets. Four subsets were used as training data, and one subset was retained as a validation set for model testing. The cross-validation process was then iterated four more times, with each of the five subsets being used as validation data exactly once. Subsequently, model performance metrics were evaluated for training and validation groups separately in each model iteration.

By separating deceased and surviving patients separately into five mortality-stratified subsets first and then combining these into the final five subsets, we maintained the same proportion of deceased and surviving patients in each of the final five subsets.

We evaluated several machine learning techniques for both models: Logistic Regression, Random Forest, Artificial Neural Network (ANN), K-Nearest Neighbors (KNN), Linear Discriminant Analysis (LDA), and Naive Bayes.

We took advantage of the Scikit-learn machine learning library to implement both preprocessing algorithms and models (Garreta and Moncecchi, 2013).

### Symptom Prediction Model

The SPM output predicts symptoms for SARS-CoV-2 positive patients. Since there are 12 symptom groups, we judged the models’ overall performance by a single metric, the prevalence-weighted mean of the twelve ROC-AUCs (Mandrekar, 2010), in which the ROC-AUCs were weighted by symptom prevalence.

### Mortality Prediction Model

The MPM calculates the probability of death for SARS-CoV-2 positive patients. Each model’s performance was measured in terms of a ROC-AUC.

## Results

### Participants

Baseline characteristics of patients and their symptoms are shown in Table 2. Of all 23,749 confirmed or suspected COVID-19 patients, 2,440 (10.27%) passed away at the end of the study (see *Discussion*). A comparison of the characteristics of survived and deceased patients is shown in Table 3. A comparison of the characteristics of patients with and without each symptom is shown in Supplementary Tables S1–S16.

**TABLE 2:** Patient characteristics and symptoms. Baseline characteristics, symptoms, and death outcomes for COVID-19 patients.

Continuous variables	
Variable	Median (±IQR)
Age	52 (±29)
Categorical/Binary variables	
Variable	Count (percent)
Sex	
Male	12,597 (53.04%)
Female	11,152 (46.96%)
Cardiovascular disease	2,471 (10.4%)
Diabetes	2,068 (8.71%)
Hypertension	2,004 (8.44%)
Respiratory diseases	546 (2.3%)
Cancer	477 (2.01%)
Kidney disorders	416 (1.75%)
Neurological disorders	264 (1.11%)
Immune disorders	178 (0.75%)
Blood disorders	152 (0.64%)
Current pregnancy	139 (0.59%)
Liver disorders	119 (0.5%)
Endocrine disorders	97 (0.41%)
Organ or bone marrow transplant	29 (0.12%)
Mental illnesses	19 (0.08%)
Thrombosis	15 (0.06%)
Past COVID-19 infection	10 (0.04%)
Outcomes	
Survived	21,309 (89.73%)
Dead	2,440 (10.27%)
Symptoms	
Cough	11,995 (50.51%)
Respiratory distress	10,342 (43.55%)
Muscular pain or fatigue	9,249 (38.94%)
Fever or chill	8,553 (36.01%)
Gastrointestinal problems	2,469 (10.4%)
Headache	1,120 (4.72%)
Chest pain	745 (3.14%)
Consciousness disorders	698 (2.94%)
Loss of smell or taste	659 (2.77%)
Vertigo	501 (2.11%)
Sore throat	157 (0.66%)
Paresis or paralysis	121 (0.51%)

**TABLE 3:** Comparison between survived and deceased patient groups. Comparative evaluation of the characteristics of survived and deceased COVID-19 patients.

Continuous variables				
Variable	Median in survivors (±IQR)	Median in deceased (±IQR)	F-test statistics	F-test *p*-value
Age	49 (±27)	70 (±21)	2,039.47	<0.001
Categorical/Binary variables				
Variable	Count in survivors (percent in survivors)	Count in deceased (percent in deceased)	Chi2 statistics	Chi2 *p*-value
Sex				
Male	11,163 (52.39%)	1,434 (58.77%)	16.82	<0.001
Female	10,146 (47.61%)	1,006 (41.23%)	19	<0.001
Cardiovascular disease	2,039 (9.57%)	432 (17.7%)	139.29	<0.001
Diabetes	1,693 (7.94%)	375 (15.37%)	138.57	<0.001
Hypertension	1,676 (7.87%)	328 (13.44%)	80.71	<0.001
Respiratory disorder**s**	462 (2.17%)	84 (3.44%)	15.47	<0.001
Cancer	343 (1.61%)	134 (5.49%)	164.28	<0.001
Kidney disorders	317 (1.49%)	99 (4.06%)	82.54	<0.001
Neurological disorders	207 (0.97%)	57 (2.34%)	36.68	<0.001
Immune disorders	152 (0.71%)	26 (1.07%)	3.62	0.057
Blood disorders	112 (0.53%)	40 (1.64%)	42.43	<0.001
Current pregnancy	133 (0.62%)	6 (0.25%)	5.35	0.021
Liver disorders	101 (0.47%)	18 (0.74%)	3.04	0.081
Endocrine disorders	88 (0.41%)	9 (0.37%)	0.1	0.747
Organ or bone marrow transplant	25 (0.12%)	4 (0.16%)	0.39	0.533
Psychiatric disorders	16 (0.08%)	3 (0.12%)	0.63	0.428
Thrombosis	13 (0.06%)	2 (0.08%)	0.15	0.696
Past COVID-19 infection	10 (0.05%)	0 (0.0%)	1.15	0.285

We used statistical hypothesis tests to demonstrate each predictor variable’s significance to the model outputs. We employed the F-test (Snedecor, 1957) technique for age, a continuous variable, and the Chi-square (Snedecor, 1957) technique for other categorical variables such as sex and PMH.

### Model Specification

We evaluated six machine learning methods for both the SPM and MPM, which are listed, together with the hyperparameters used in Table 4.

**TABLE 4:** Machine learning methods and hyperparameters used.

The Mortality Prediction Model		
Method	Parameter	Value(s)
Logistic Regression	C	1.0
Random Forest	Number of trees	500
	Min. Number of samples at a leaf node	%0.1 of all samples
	Criterion	Gini
Artificial Neural Networks	Number of layers	3
	Output space dimensionality for each layer	32, 16, 1
	Activation function for each layer	Tanh, tanh, sigmoid
K-Nearest Neighbors	K	10
	Weight function	Distance
Linear Discriminant Analysis	Solver	SVD
Naive Bayes	Interval size of age categories	0.1
The Symptom Prediction Model		
Method	Parameter	Value
Logistic Regression	C	1.0
Random Forest	Number of trees	200
	Min. Number of samples at a leaf node	%0.1 of all samples
	Criterion	Gini
Artificial Neural Network	Number of layers	4
	Output space’s dimensionality for each layer	32, 32, 32, 12
	Activation function for each layer	Tanh, tanh, tanh, tanh, sigmoid
K-Nearest Neighbors	K	5
	Weight function	Distance
Linear Discriminant Analysis	Solver	SVD
Naive Bayes	Interval size of age categories	0.1

### Model Performance

#### Symptom Prediction Model

**FIGURE 1:**
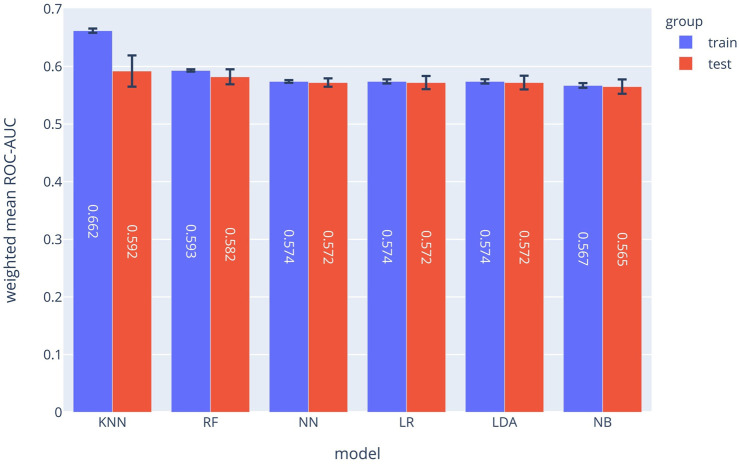
Prevalence-weighted means ROC-AUCs for different ML models. The models were used to implement the Symptom Prediction Model (SPM). Error bars denote the standard deviation over different cross-validation folds.

The SPM can be considered as 12 separated classifiers; each predicts the occurrence of a specific symptom. While the performance of each sub-classifier can be evaluated separately, the overall performance can be assessed using the prevalence-weighted mean of the ROC-AUCs, since the symptoms have different prevalence. The prevalence-weighted mean ROC-AUC for each method is illustrated in Figure 1. Although the KNN method provided the highest weighted mean ROC-AUC for the test data, it was the least robust method since its performance varied considerably for different validation folds (note standard deviation bars). The Random Forest method achieved better overall performance and robustness. The weighted mean ROC-AUC value of this method was 0.582 for the test data.

Moreover, the performance of the SPM can be evaluated for each symptom separately. The ROC-AUC values for predicting consciousness disorder, paresis or paralysis, and chest pain were 0.785, 0.729, and 0.686, respectively. Also, at a specificity of 70%, the sensitivities were 73%, 50%, and 53%, respectively.

As shown in Figure 1, the random forest model with a mean ROC-AUC of 0.8 and 0.79 has the highest efficiency in the training and the validation groups, respectively, followed by the Neural Network and LDA. In the symptom prediction model, the ROC-AUC values of all models in addition to the weighted average of ROC-AUC of different ML methods for each symptom are shown in Supplementary Figure S1. Supplementary Figure S2 delineates each method's performance for all symptoms as a Radar chart.

Based on the ROC diagram and the information from the database, the other performance metrics of the other models were identified. In addition to the ROC-AUC of the risk prediction model, we calculated the sensitivity and the negative and positive predictive value (NPV and PPV respectively) for each model. The detailed results of all six algorithms for both MPM and SPM are shown in Supplementary Tables S17 and S18.

The calibration plot of the RF implementation for each symptom predictor (sub-classifier) is depicted in Supplementary Figure S3 which shows the calibration plot of the RF implementation of each symptom.

### Mortality Prediction Model

**FIGURE 2:**
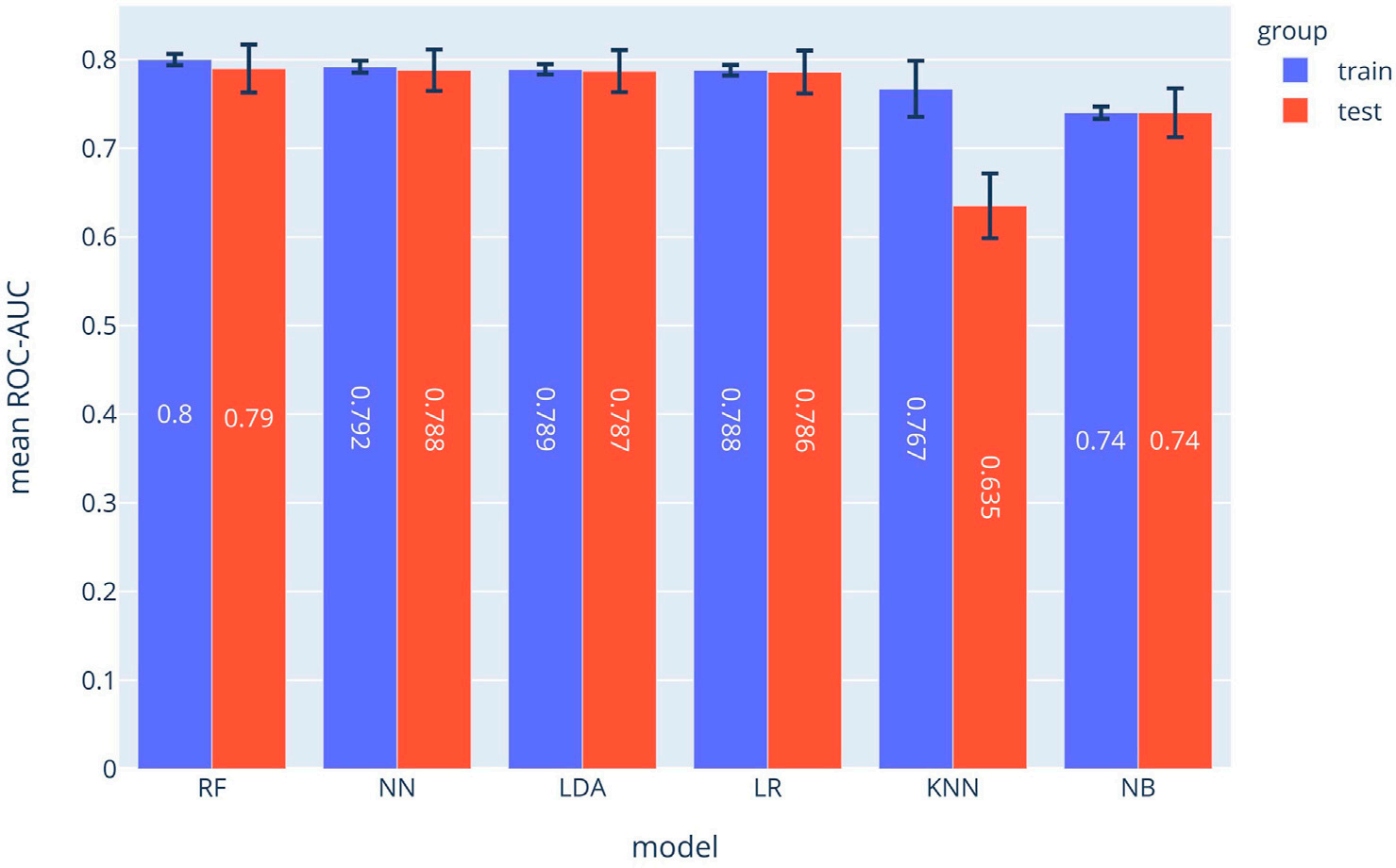
ROC-AUCs of different ML models which were used to implement the MPM. The Random Forest (RF) model outperformed the other approaches.

The ROC-AUC values for each method are depicted in Figure 2. In the MPM classifier, the Random Forest method outperformed the other methods just as for the SPM. The achieved ROC-AUC value was 0.79 for the test data.

Supplementary Figure S4 shows ROC diagrams representing the true-positive rates vs. false-positive rates for each method used to implement the MPM. The calibration plot of the RF model is depicted in Supplementary Figure S5. Calibration indications such as Mean Calibration Error are also shown in the Supplementary Figure S5 for different methods.

### Model Input-Output Correlations

We used the Chi-square test and the F-test to evaluate the extent to which PMH, sex, or age predict the outputs of the SPM and MPM. The larger the values of these test values are for each predictor variable, the more the predictor variable is predictive of the output of the models. For categorical predictor variables (i.e., PMH and sex), the Chi-square hypothesis test was used. To evaluate the predictive value of a categorical variable, we examined whether it was more common in patients who died (MPM) or in patients with a particular symptom (SPM). For the only continuous variable (age), we used the F-test. To find the impact of age, we examined if the age median was higher in dead patients (MPM) or patients with a particular symptom (SPM).

For the SPM model, Supplementary Figure S6 shows how each factor in the PMH was correlated with each symptom using the Chi-square test. For example, patients with diabetes or cardiovascular disease were more likely to have consciousness disorders and chest pain in case of infection with COVID-19. The effect of age on each symptom is shown in Supplementary Figure S7 using the F-test. Older patients were more likely to develop symptoms such as respiratory distress and consciousness disorder but also less likely to develop symptoms such as muscular pain or fatigue.

In addition, for the MPM, the impact of each PMH on death is shown in Supplementary Figure S8. In our analysis, cancer, cardiovascular disease, and diabetes have the greatest effects on the risk of death in patients with Covid 19; on the other hand, pregnancy or being female decreased the chances of death. The F-test statistic of age in the MPM model is 2,039.47, which explains the increase in mortality risk from aging.

DeLong's test shows the statistically significant difference between AUCs of models. The DeLong tests for the MPM and SPM predictions are shown through Supplementary Figures S9–S21.

### Validation of the Model for Each Mortality Peak

**FIGURE 3:**
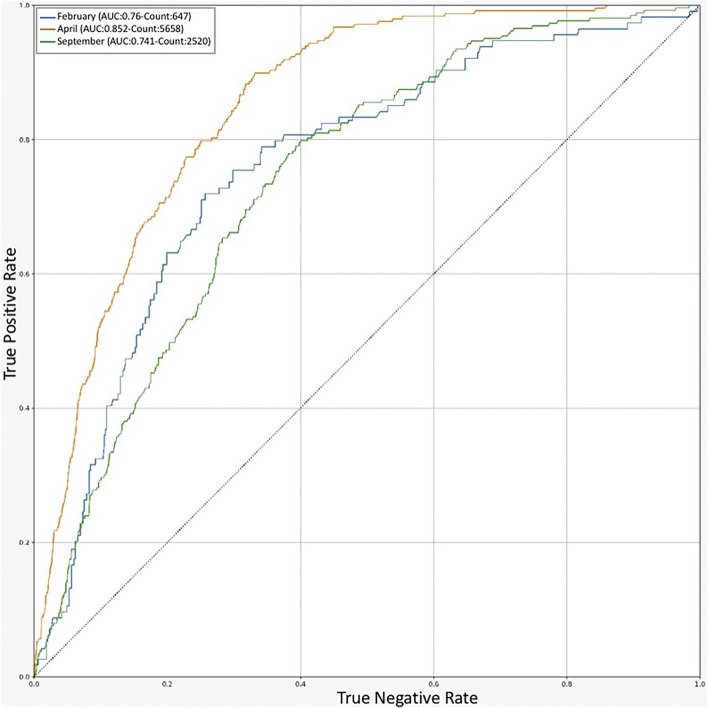
ROC chart for Prediction of RF models in different timelines**.** An indicator of model performance on validation dataset in the peak months of COVID-19 outbreak in Iran.

For additional validation of our model, we evaluated the performance of the final random forest for MPM during the periods with the highest rate of mortality. The data corresponding to each available mortality peak (april, February, and September 2020) was selected from the validation dataset of each model, and the outcome (recovery or death of the patient) during each period was predicted by the model and shown as a ROC diagram (Figure 3). Despite the variation of the AUC in the mortality peaks, the weighted average of the AUC values corresponding to each period was approximately equal to the average model yield for the entire data. We can conclude that the model continues to perform equally well during each mortality peak. The cause of the high yield in april could be explained by the large number of available samples which would allow the algorithm to learn more accurately.

## Discussion

Our objective in this study was to develop 2 ML models to predict the mortality and symptoms of COVID-19-positive patients among the general population using age, gender, and comorbidities alone. These models can guide the design of measures to combat the COVID-19 pandemic. The prediction of vulnerability using the models allows people in different risk groups to take appropriate actions if they contract COVID-19. For example, people who fall into the low-risk group can start isolation sooner when the predicted symptoms appear and refer to a hospital only if the symptoms persist. As a result, the risk of disease spread and the pressure on the health care system from unnecessary hospital visits, costs, and psychological and physical stress to the medical staff could be reduced (Emanuel et al., 2020). In contrast, people who are predicted to be at higher risk are recommended to seek medical care immediately. Predictions can speed up the treatment process and ultimately decrease mortality.

Our study has shown that multiple symptoms have strong correlations with different medical history factors. Symptoms can be either amplified or attenuated by health backgrounds; for instance, hypertension, diabetes, and respiratory and neurological disorders increased the chances of loss of smell or taste; however, pregnancy, cancer, higher age, cardiovascular disease, and liver, immune system, blood, and kidney disorders have attenuated the appearance of this symptom.

Due to the complexity of the COVID-19 pathogenesis, many clinical studies revealed contradictory results, for example, the effectiveness or ineffectiveness of remdisivir (Beigel et al., 2020; Goldman et al., 2020; Wang et al., 2020). We hypothesize that the imbalance of mortality risks between the intervention and control groups could have been a problem in these studies. With the help of our model, such problems could be partially solved by equalizing the mortality baseline in different clinical groups.

Our AI models can also be beneficial for COVID-19 vaccine testing and prioritization strategies. The limited number of approved vaccines in the first months of the vaccination process and the potential shortages make vaccine prioritization inevitable. This prioritization would be more important for developing countries that do not have the resources to pre-order vaccines from multiple companies (Persad et al., 2020). Having a mortality prediction tool for each individual could be a valuable tool for governments to decide on vaccines' allocation.

### Limitations

Since our dataset was collected by the HIS, it did not contain COVID-19 patients that did not refer to a hospital or had no major symptoms to be identified as infected. This could explain the high mortality rate in our and other studies (Fumagalli et al., 2020; Gue et al., 2020; Yadaw et al., 2020). However, for a systematic study with few confounding variables, uniform data collection is essential, which can only be realistically ensured with hospital data.

Also, other variables such as the viral load may be important but are difficult to measure and are not readily available. We opted for easily accessible predictor variables to allow the widespread use of the models.

One way to improve the models is to subgroup-specific factors in the medical history or specific symptoms further. The main reason for grouping factors and symptoms was the low prevalence of certain subsets in the dataset.

In conclusion, we evaluated 15 parameters (Table 1) for predicting the symptoms and the mortality risk of COVID-19 patients. The ML models trained in this study could help people quickly determine their mortality risk and the probable symptoms of the infection. These tools could aid patients, physicians, and governments with informed and shared decision-making.

## Data Availability

The original contributions presented in the study are included in the article/Supplementary Material, further inquiries can be directed to the corresponding authors.

## Abbreviations

AI, artificial intelligence; COVID-19, coronavirus disease of 2019; intensive care unit, IQR; interquartile range, KS; Kolmogorov-Smirnov, LR; logistic regression, ML; machine learning, RF; random forest, RDW; red blood cell, distribution width; ROC, receiver operating characteristic; HIS, hospital information system.
